# Hearing conservation in the primary aluminium industry

**DOI:** 10.1093/occmed/kqv168

**Published:** 2015-10-15

**Authors:** A. M. Donoghue, N. Frisch, C. Dixon-Ernst, B. J. Chesson, M. R. Cullen

**Affiliations:** ^1^Alcoa of Australia, PO Box 252, Applecross, Perth, WA 6953, Australia,; ^2^Alcoa Corporate Centre, 201 Isabella Street, Pittsburgh, PA 15212-5858, USA,; ^3^Occupational Hygiene Solutions Pty Ltd, 26 Leake Street, Peppermint Grove, Perth, WA 6011, Australia,; ^4^Division of General Medical Disciplines, Stanford University School of Medicine, 1265 Welch Road, MSOB X-338, Stanford, CA 94305-5411, USA.

**Keywords:** Audiometry, hearing loss, minerals processing, mining, noise, noise-induced hearing loss, smelter.

## Abstract

**Background:**

Noise-induced hearing loss has been an intractable problem for heavy industry.

**Aims:**

To report our experience in reducing the incidence of age-corrected confirmed 10 dB hearing shifts (averaged over 2, 3 and 4kHz) in employees in the primary aluminium industry in Australia over the period 2006–13.

**Methods:**

We analysed annual audiometric data to determine the number of permanent hearing shifts that occurred in employees in two bauxite mines, three alumina refineries and two aluminium smelters. Annual hearing shift rates were calculated based on the number of employees tested per year. Hearing conservation initiatives undertaken during the study period are described. An assessment of similar exposure group noise exposures was also undertaken to determine the magnitude of noise exposure reduction during the study period.

**Results:**

Across all operations, hearing shift rates declined from 5.5% per year in 2006 to 1.3% per year in 2013 (*P* < 0.001). The decline in shift rates was greater in mines and refineries, where baseline shift rates were higher, than in smelter workers. Modest reductions in noise exposure occurred during the study period.

**Conclusions:**

We observed a substantial decline in hearing shift rates during the study period. We describe the hearing conservation initiatives that were collectively associated with this decline. We suspect these initiatives could be deployed relatively easily and at modest cost in other industries with noise-exposed employees.

## Introduction

Exposure to noise in industry is ubiquitous and noise-induced hearing loss (NIHL) has been an intractable problem for heavy industry. For example, in the USA, ~22 million workers are exposed to hazardous noise levels and occupational hearing loss is one of the most common work-related diseases [1]. The prevalence of hearing impairment has remained fairly constant at ~20% in US industry over the last 30 years [2]. The incidence of hearing impairment has fallen by almost 50% in that time, suggesting that hearing conservation efforts are probably having some effect [2]. Nevertheless, there is clearly room for further noise control and more effective hearing conservation programmes. Recent advances in technology have enabled quantitative fit testing of hearing protection in individual workers [3]. This may prove to be a useful addition to hearing conservation programmes, by improving use of effective hearing protection.

We report our experience reducing the incidence of age-corrected confirmed 10 dB hearing shifts among employees in the primary aluminium industry in Australia, including bauxite mining, alumina refining and aluminium smelting operations, over the period 2006–13. During this period, the employer (Alcoa of Australia) introduced a number of new initiatives, including quantitative fit testing of hearing protection.

## Methods

At the start of the study period, the mandated elements of Alcoa’s global health standard on hearing conservation had already been deployed for several years. This standard requires annual audiometry for personnel whose noise exposure for 12 or more days per year equals or exceeds 85 dB(A)Leq8h, a 15-min short-term exposure limit of ≥100 dB(A) or a sound pressure level of 140 dB linear peak [4]. Audiometry is also required at a minimum of every 3 years for personnel whose noise exposure for 12 or more days per year equals or exceeds 82 dB(A)Leq8h but is <85 dB(A)Leq8h. Noise exposure assessments, including sound level surveys and personal dosimetry, are required where noise exposures may equal or exceed the values above. Hearing conservation training and hearing protection are required for all employees meeting the noise exposure criteria for audiometry.

Recommended training includes education on the potential harmful effects on hearing of exposure to noise; an explanation of the location-specific hearing conservation and engineering noise control programme; an explanation of hearing protectors, including the advantages and disadvantages of various types, the attenuation provided by various types of hearing protectors, instructions on their selection, fitting, use, and care and a discussion of how they are chosen to offer optimal protection from the effects of the noise in the location-specific work environment. Additionally, the purpose of audiometric testing and an explanation of the audiometric test procedures with emphasis on the importance of participation as a way of detecting early signs of hearing loss to prevent hearing impairment is provided.

Audiometry in the Australian operations is conducted under the supervision of the chief medical officer at on-site medical centres by occupational health nurses certified in audiometric surveillance using audiometers and hearing booths tested and certified annually. Audiometric data are uploaded to the occupational health manager (OHM) medical records database. The US Occupational Safety and Health Administration (OSHA) criteria for classifying hearing shifts are applied to the audiometric results [5]. An age-corrected 10 dB hearing shift, as defined by OSHA, is a pure tone audiometric threshold decrement of 10 dB or greater from the last baseline averaged across the frequencies of 2, 3 and 4kHz, with age correction applied to the result. The employee’s baseline is established by either the audiogram taken on commencing employment or the audiogram at the time of the last age-corrected confirmed 10 dB hearing shift if there has been one in the same ear. Age correction is undertaken to allow for presbyacusis [6]. Any age-corrected 10 dB hearing shift requires a repeat test undertaken within the next 30 days after a noise-free interval of 14h to exclude temporary threshold shift. If the retest confirms the shift, it is referred to as an age-corrected confirmed 10 dB hearing shift.

For this study, age-corrected raw audiometric data were anonymized prior to export from the OHM database. Data for 2006–13 inclusive were imported into Excel for analysis. For each of the mines, refineries and smelters, the number of age-corrected confirmed 10 dB hearing shifts was determined for each year and divided by the number of employees undergoing audiometry in the same year. This figure multiplied by 100 gave the age-corrected confirmed 10 dB hearing shift rate (% per year). The shift rate is not quite the percentage of employees with a hearing shift in a given year, as some individuals may have had two hearing shifts (one in each ear) in a given year. Unpublished research by Yale University has previously estimated the age-corrected confirmed 10 dB hearing shift rate in employees not exposed to occupational noise as 1% per year [P. M. Rabinowitz, D Galusha (personal communication)]. If this ‘background’ shift rate could be attained in noise-exposed employees, occupational NIHL would have been minimized. Alcoa therefore adopted a target age-corrected confirmed 10 dB hearing shift rate of 1% per year for employees exposed to occupational noise, to be achieved by the year 2020. ‘Background’ hearing shifts are due to non-occupational noise exposures (for example loud music, weapons firing and motor sports) and a variety of other causes of sensorineural hearing loss (infectious diseases, pharmacologic agents, head injury, therapeutic radiation exposure, neurological disorders, cerebrovascular disorders, immune disorders, bone disorders, central nervous system neoplasms and Ménière’s disease) [7].

From 2006 to 2013, Alcoa of Australia introduced a range of initiatives in addition to the mandated elements of hearing conservation outlined above (see Box 1).

**Box 1**

These included

1. Holding noise summit meetings and health promotion campaigns to motivate and educate employees about the cause and consequences of noise-induced hearing loss (NIHL).
2. Producing a DVD on NIHL called ‘The Road to Silence’.
3. Instituting the FitCheck (Michael and Associates Inc) insert-type hearing protector measurement system to quantify earplug noise attenuation in each employee [8]. This was undertaken every 2 years and enables education of each employee on optimum fit technique and earplug selection. In 2014, the 3M^™^ E-A-RFit^™^ equipment and fit testing programme was adopted in Australia [9], when updated FitCheck became unavailable.
4. Provision of a broad range of hearing protection devices.
5. Adoption of non-age-corrected 10 dB hearing shifts as an early warning of an impending age-corrected confirmed 10 dB hearing shift [10]. Employees with a non-age-corrected shift have a personal hearing conservation review. Temporary threshold shifts (age-corrected non-confirmed 10 dB hearing shifts) are also used as a trigger for a personal review.
6. Bimonthly audits to ensure hearing protectors are fitted and worn properly.
7. Initiation of visual walkway signage indicating the need for hearing protection in noise-exposed areas of the operations.
8. Use of personalized hard hat stickers to remind employees which type of earplug obtained good attenuation during FitCheck testing.
9. Requirements that all operating centres identify engineering noise control projects and complete them when practicable.
10. Instituting a ‘Buy Quiet’ approach to procurement of new equipment to ensure noise levels are considered when selecting new products.
11. Introduction of personal noise indicator badges (3M^™^ Noise indicator NI-100) with green or red flashing lights to indicate the real-time noise level is below or above 85 dB(A) [11].
12. Use of noise dosimeters to ensure confirmation audiograms are done after a documented 14h noise-free interval.

To determine the extent of noise reduction during the study period, we examined occupational hygiene data on noise monitoring. Noise exposures were assessed for all similar exposure groups (SEGs) across all locations. SEGs are defined as ‘groups of workers having the same general exposure profile because of the similarity and frequency of the tasks they perform, the materials and processes with which they work and the similarity of the way they perform their tasks’ [12]. Noise exposures were classified as ‘unacceptable’ if the exposure for a SEG had a >5% probability of exceeding the occupational exposure limit (OEL) on any day. Alcoa set specific noise exposure reduction targets to be completed over 3-year cycles. The 2006–08 cycle target was to achieve a 20% reduction in each location in unacceptable noise exposures (measured as either a reduction in noise magnitude or as a reduction in the number of unacceptable SEGs) through engineering projects and design changes. In the 2009–11 cycle, following the global financial crisis, capital expenditure was constrained and the target was modified to one where each location specified engineering projects deemed achievable and then attempted to complete 100% of these projects over the 3-year period. Post 2011, the noise exposure reduction targets were suspended due to further constraints on capital projects.

We assessed the impact of the engineering noise reduction programmes on noise exposure at each location for each of the 3-year cycles. For each unacceptable SEG, the individual noise exposure data points [recorded as dB(A) values] within the SEG were converted to linear metric values reflecting the ratio of the noise exposures to the OEL (expressed as percentages of the OEL). The arithmetic mean of these percentages of OEL values was taken to summarize noise exposure within each unaccept able SEG. To assess the overall noise exposure at each location, the geometric mean of all of the summary unacceptable SEG values at each location was then calculated for the beginning of the 3-year metric cycle (baseline) and for the end of the cycle. The difference between baseline and end of cycle (expressed as a percentage of baseline) was taken to be the noise reduction achieved at that location. The noise reduction results were then averaged across all locations (weighted by the baseline number of unacceptable SEGs at each location) to determine the overall reduction across the Australian organization. The chi-squared test for trend was used to determine the statistical significance of reductions in hearing shift rates over the study period [13]. Stanford Institutional Review Board approval for epidemiological studies using anonymized data was obtained for this study.

## Results

In 2011, there were 865 employees in the mines, 2994 in refineries and 1246 working in smelters. Audiometric surveillance was required for 796 mining employees (92%), 2631 refinery employees (88%) and 1091 smelter employees (88%) exposed to noise. Table 1 is an example from the smallest location of the noise reduction calculations undertaken for the ‘unacceptable’ SEGs at each location, as described in the Methods section. Annual data on audiometric testing numbers, age-corrected confirmed 10 dB hearing shifts and the corresponding percentage shift rates are shown in Table 2.

**Table 1.** Example of noise reduction calculations for one small location (Anglesea Power Station ‘unacceptable’ SEGs) during the 2006–08 metric cycle

SEG ID	SEG shift length (h)	OEL [dB(A)]	SEG baseline [dB(A)]	SEG baseline exposure as % of OEL		SEG end of cycle [dB(A)]	SEG end of cycle exposure as % of OEL	Reduction % from baseline
A08	12	83	84.4	138		85.0	159	−21
A09	10	84	87.1	205		86.8	191	14
A17	10	84	82.8	76		82.6	72	4
A19	10	84	82.3	68		81.9	62	6
A20	10	84	87.7	235		84.0	100	135
A21	10	84	84.7	118		84.5	112	6
A22	10	84	101.2	5311		99.9	3934	1377
A23	10	84	84.3	107		84.7	118	−11
A31	12	83	84.8	152		86.9	246	−94
	Baseline GM % OEL [Equivalent to 86.8 dB(A)]			192	End of cycle GM % OEL [Equivalent to 86.5 dB(A)]		177	(8% reduction)

GM, geometric mean. 3 dB doubling rate used in the calculations. dB(A) equivalents were calculated from GM % OEL values assuming a 10h shift length.

**Table 2.** Age-corrected confirmed 10 dB hearing shift data for each year of the study in the mines, refineries and smelters

	2006	2007	2008	2009	2010	2011	2012	2013
Mines								
Hearing shifts (*n*)	49	68	30	35	18	11	12	10
Employees tested (*n*)	727	755	837	801	804	782	770	766
Hearing shift rate (%)	6.7	9.0	3.6	4.4	2.2	1.4	1.6	1.3
Refineries								
Hearing shifts (*n*)	167	111	70	81	89	47	23	31
Employees tested (*n*)	2619	2769	2777	2691	2586	2602	2409	2430
Hearing shift rate (%)	6.4	4.0	2.5	3.0	3.4	1.8	1.0	1.3
Smelters								
Hearing shifts (*n*)	37	40	25	36	19	12	18	14
Employees tested (*n*)	1290	1254	1268	1197	769	1110	1016	1071
Hearing shift rate (%)	2.9	3.2	2.0	3.0	2.5	1.1	1.8	1.3

**Figure 1.**
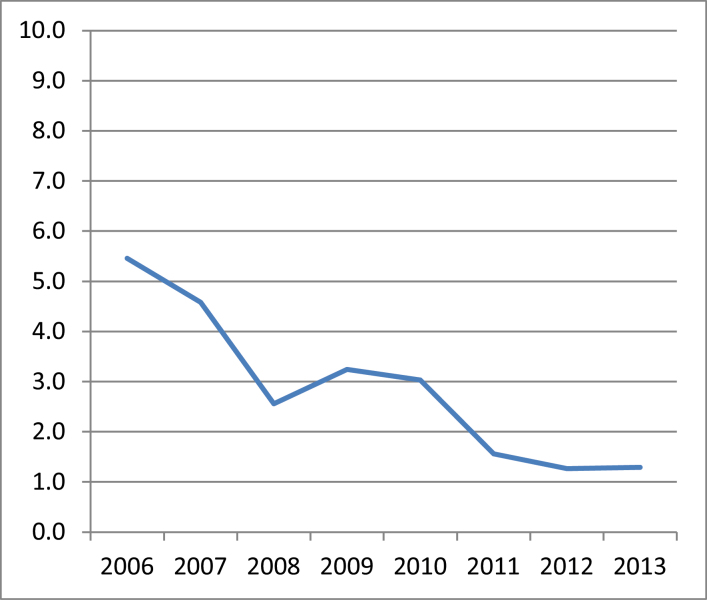
Age-corrected confirmed 10 dB hearing shift rate (% per year) for all of Alcoa of Australia’s mines, refineries and smelters combined. Trend *P* < 0.001.

**Figure 2.**
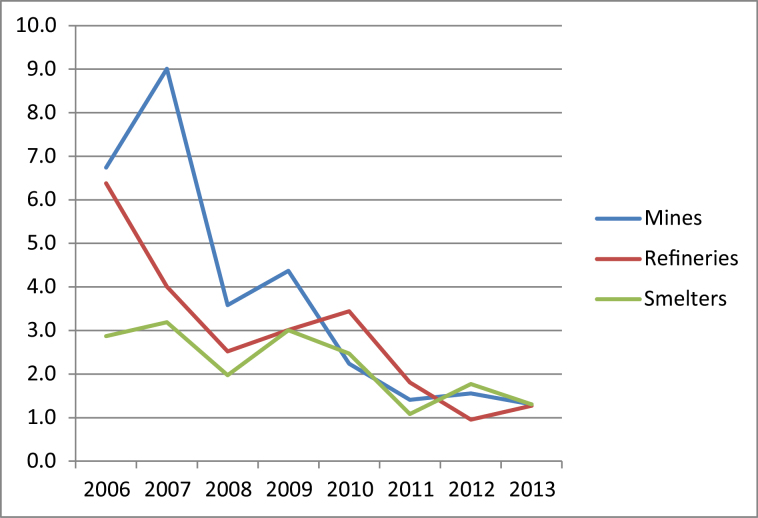
Age-corrected confirmed 10 dB hearing shift rate (% per year) for Alcoa of Australia’s mines, refineries and smelters viewed separately. Trend: Mines *P* < 0.001, Refineries *P* < 0.001, Smelters *P* < 0.05.

When all of the operations were considered in aggregate, there was a statistically significant decline in the age-corrected confirmed 10 dB shift rate, from 5.5% per year in 2006 to 1.3% per year in 2013 (Figure 1). The declines in shift rates were particularly marked for the mines and refineries, which started at higher baseline shift rates than the smelters (Figure 2). All declines were statistically significant. The shift rate remained below 2% per year for the mining, refining and smelting operations throughout the three most recent years of 2011–13, indicating a degree of consistency and durability.

Table 3 shows unacceptable noise SEG data over each of the 3-year cycles (2006–08 and 2009–11) for each mine, refinery and smelter. Over the 2006–08 cycle, an overall 10% reduction in noise level magnitude was achieved for all SEGs that had been classified as unacceptable. This is equivalent to a reduction in average noise level from 88 to 87.6 dB(A). For the 2009–11 cycle, an overall reduction in noise level magnitude of 8% was achieved, equivalent to a reduction in average noise level from 85.5 to 85.1 dB(A).

**Table 3.** Noise reductions calculated for all locations ‘unacceptable’ SEGs during the 2006–08 and 2009–11 metric cycles

Noise reduction metric	APS	KWI	PIN	PTH	PTL	WAM	WGP	All locations
2006–08 metric cycle								
Baseline ‘unacceptable’ noise SEGs (*n*)	9	15	81	35	44	24	75	283
Baseline GM % OEL	192	476	372	287	308	337	253	
End of cycle GM % OEL	177	442	353	246	223	311	244	
Reduction from baseline (%)	8	7	5	14	27	8	4	10
Baseline equivalent [dB(A)]	86.8	90.8	89.7	87.6	87.9	88.3	88.0	88.0
End of cycle equivalent [dB(A)]	86.5	90.4	89.5	86.9	86.5	87.9	87.9	87.6
Based on shift length (h)	10	10	10	12	12	12	10	12
2009–11 metric cycle								
Baseline ‘unacceptable’ noise SEGs (*n*)	8	133	114	45	46	43	55	444
Baseline GM % OEL	191	141	175	229	224	175	196	
End of cycle GM % OEL	238	128	158	198	214	157	186	
Reduction from baseline (%)	-25	10	10	13	4	11	5	8
Baseline equivalent [dB(A)]	86.8	85.5	86.4	86.6	86.5	85.4	86.9	85.5
End of cycle equivalent [dB(A)]	87.8	85.1	86.0	86.0	86.3	84.9	86.7	85.1
Based on shift length (h)	10	10	10	12	12	12	10	12

APS, Anglesea Power Station; GM, geometric mean; KWI, Kwinana Refinery; PIN, Pinjarra Refinery; PTH, Point Henry Smelter; PTL, Portland Smelter; WAM, Western Australian Mining; WGP, Wagerup Refinery. 3 dB doubling rate used in the calculations. dB(A) equivalents were calculated from GM % OEL values assuming the stated shift lengths.

## Discussion

The 2013 results for the worksites reported here approximate the ‘background’ 1% per year shift rate found previously in non-noise-exposed occupations within the aluminium industry in the USA [P. M. Rabinowitz, D Galusha (personal communication)]. This suggests occupational NIHL has been minimized in the worker population under surveillance here.

Given the descriptive nature of the study, we are not able to state which of the initiatives contributed to the improvement in hearing shift rates. We posit that those associated with improving the knowledge of the workforce about NIHL and particularly about the importance of correctly wearing hearing protection had the greatest impact. The noise summits organized in 2006 and 2007 were conducted across all operations and enabled a core body of employees to become champions for hearing conservation. The summits included a specially produced educational DVD, ‘The Road to Silence,’ in which employees with NIHL explained how it had affected their lives. This was followed by other campaigns, in particular, a programme to fit test all noise-exposed employees to ensure they understood correct fitting of earplugs or earmuffs. This fit testing programme found that many employees had not previously been wearing hearing protection effectively and was continued on a biannual basis for all noise-exposed employees. Annual audiometric testing for all noise-exposed employees and personal hearing conservation reviews for those with early NIHL helped to identify the most significant noise risks in the employee’s area as well as assist the worker with their own hearing protection management.

Although it is likely that the initiatives described above have collectively brought about the observed decline in hearing shift rates, we also considered potential confounders. There were modest changes in turnover of the workforce during the study period. The percentage turnover of employees each year during the period 2006–13 ranged from 5 to 10% in the mines, from 8 to 12% in the refineries and from 4 to 9% in the smelters. It seems unlikely that changes of these magnitudes would materially impact hearing shift rates. There were no meaningful changes in the average hours worked per week. During the period 2008–13, these ranged from 35.7 to 36.0 in the mines, from 35.4 to 36.5 in the refineries and from 40.8 to 41.0 in the smelters. There were modest changes in production levels: bauxite production increased from 30996 kt in 2006 to 34181 kt in 2013 (10%); alumina production increased from 8224 kt in 2006 to 9182 kt in 2013 (12%) and aluminium production decreased from 530 kt in 2006 to 488 kt in 2013 (−8%). It seems unlikely that increases in production in mining and refining would lead to reduced noise exposures and contribute to the observed decline in hearing shift rates. Although it seems more likely that the decline in aluminium production might have contributed to the observed effect in smelters, the decline was modest (8%) and the exposure data for unacceptable SEGs probably give a more useful insight.

We also considered the possibility that the decline in confirmed hearing shift rates might be due to a decline in compliance with recall for confirmation audiometry. As the OHM database is set to automatically confirm hearing shifts detected on a first test if the person is not re-tested within 90 days, non-compliance with re-testing could not have contributed to the figures we present.

There was a significant change in the average noise levels between the two cycle periods shown in Table 3. This was because the noise reduction metric was based on success in reducing noise exposures for unacceptable SEGs that had been identified at the start of the metric cycle only. During the 3-year cycles, each location was required to continue noise dosimetry to update exposure data for their SEGs. Any new unacceptable SEGs were added to an updated list. In some locations,

this led to an increase in the number of unacceptable SEGs, particularly in the 2006–08 cycle. This was especially so at Kwinana Refinery where reorganization of work groups led to an increased number of smaller work groups. In Table 3, the number of unacceptable noise SEGs at Kwinana rose from 15 at the start of the 2006 cycle to 138 by the end of this cycle, and for the Australian organization as a whole the number increased from 283 to 458. While many of these new SEGs were identified as having unacceptable noise exposure, in many cases their noise exposures were significantly lower than the earlier identified unacceptable SEGs. In some cases, these newly identified SEGs were borderline unacceptable. This had the impact of lowering the average unacceptable noise level from one cycle to the next.

While the overall noise reductions appear to be modest, we believe that these efforts would have had some impact in reducing NIHL, although probably less so than the improved use of hearing protection. Apart from the reduction in exposure experienced by some SEGs, the increased focus on identifying noise sources also focused attention on the need to wear hearing protection near loud plant and equipment. Unfortunately, for some SEGs, the noise levels increased, possibly as a result of ageing equipment or reduced maintenance, countering the impact of improvements in other SEGs. The modest results indicate that even with a high degree of focus, engineering reductions in noise exposures are difficult and expensive.

In summary, we report a substantial decline in the incidence of age-corrected confirmed 10 dB hearing shifts among employees in the primary aluminium industry. We have detailed the initiatives collectively associated with this decline and suspect they could be deployed easily and at modest cost in other industries. More research could define which of the initiatives are most effective.

**Key points**

- Enhanced hearing conservation initiatives in the primary aluminium industry were associated with a decline in age-corrected confirmed 10 dB hearing shift rates from 5.5% per year in 2006 to 1.3% per year in 2013.
- We posit the most important initiatives were improved education of the workforce and quantitative fit checking of hearing protection attenuation.
- Modest reductions in noise exposures occurred during the study period.

## Funding

Alcoa Inc.

## Conflicts of interest

A. M. D. is employed full time by Alcoa of Australia. N. F. and B. J. C. were previously employed by Alcoa of Australia. C.D.-E. was previously employed by Alcoa Inc. M. R. C. receives salary support from an Alcoa grant.
